# Race and Ethnicity–Adjusted Age Recommendation for Initiating Breast Cancer Screening

**DOI:** 10.1001/jamanetworkopen.2023.8893

**Published:** 2023-04-19

**Authors:** Tianhui Chen, Elham Kharazmi, Mahdi Fallah

**Affiliations:** 1 Department of Cancer Prevention, Zhejiang Cancer Hospital, Hangzhou, China; 2 Institute of Basic Medicine and Cancer, Chinese Academy of Sciences, Hangzhou, China; 3 Risk Adapted Prevention Group, Division of Preventive Oncology, German Cancer Research Center (DKFZ), Heidelberg, Germany; 4 Center for Primary Health Care Research, Lund University, Malmö, Sweden; 5 Institute of Primary Health Care, University of Bern, Bern, Switzerland; 6 Department of Global Public Health and Primary Care, University of Bergen, Bergen, Norway

## Abstract

**Importance:**

Breast cancer (BC) is the second leading cause of cancer death in women, and there is a substantial disparity in BC mortality by race, especially for early-onset BC in Black women. Many guidelines recommend starting BC screening from age 50 years; however, the current one-size-fits-all policy to start screening all women from a certain age may not be fair, equitable, or optimal.

**Objective:**

To provide race and ethnicity–adapted starting ages of BC screening based on data on current racial and ethnic disparities in BC mortality.

**Design, Setting, and Participants:**

This nationwide population-based cross-sectional study was conducted using data on BC mortality in female patients in the US who died of BC in 2011 to 2020.

**Exposures:**

Proxy-reported race and ethnicity information was used. The risk-adapted starting age of BC screening by race and ethnicity was measured based on 10-year cumulative risk of BC-specific death. Age-specific 10-year cumulative risk was calculated based on age group–specific mortality data without modeling or adjustment.

**Main Outcomes and Measures:**

Disease-specific mortality due to invasive BC in female patients.

**Results:**

There were BC-specific deaths among 415 277 female patients (1880 American Indian or Alaska Native [0.5%], 12 086 Asian or Pacific Islander [2.9%], 62 695 Black [15.1%], 28 747 Hispanic [6.9%], and 309 869 White [74.6%]; 115 214 patients died before age 60 years [27.7%]) of any age in the US in 2011 to 2020. BC mortality per 100 000 person-years for ages 40 to 49 years was 27 deaths in Black females, 15 deaths in White females, and 11 deaths in American Indian or Alaska Native, Hispanic, and Asian or Pacific Islander females. When BC screening was recommended to start at age 50 years for all females with a 10-year cumulative risk of BC death of 0.329%, Black females reached this risk threshold level 8 years earlier, at age 42 years, whereas White females reached it at age 51 years, American Indian or Alaska Native and Hispanic females at age 57 years, and Asian or Pacific Islander females 11 years later, at age 61 years. Race and ethnicity–adapted starting ages for Black females were 6 years earlier for mass screening at age 40 years and 7 years earlier for mass screening at age 45 years.

**Conclusions and Relevance:**

This study provides evidence-based race-adapted starting ages for BC screening. These findings suggest that health policy makers may consider a risk-adapted approach to BC screening in which individuals who are at high risk are screened earlier to address mortality due to early-onset BC before the recommended age of mass screening.

## Key Points

**Question:**

What are the optimal race and ethnicity–specific starting ages to initiate breast cancer screening to address racial disparity in mortality?

**Findings:**

This cross-sectional study was conducted among the total 415 277 breast cancer deaths in female patients in the US from 2011 to 2020. Study findings suggested that when breast cancer screening was recommended to start at age 50 years for the general female population, Black females should start screening 8 years earlier, at age 42 years, whereas White females could start at age 51 years, American Indian or Alaska Native and Hispanic females at age 57 years, and Asian or Pacific Islander females at age 61 years.

**Meaning:**

These findings suggest that health policy makers and clinicians could consider an alternative, race and ethnicity–adapted approach in which Black female patients start screening earlier.

## Teaser

This cross-sectional study investigates race and ethnicity–adjusted starting ages for breast cancer screening based on racial and ethnic breast cancer mortality disparities in the US.

## Introduction

In women, breast cancer (BC) is the most commonly diagnosed cancer, the second leading cause of cancer death in the US, and the deadliest cancer in 4 southern and 2 midwestern US states and globally.^1,2^ BC screening from age 50 years was associated with a reduction in BC mortality.^3^ The US Preventive Services Task Force also recommends starting BC screening from age 50 years.^4^ There is a substantial disparity in BC mortality between Black and White individuals, and this disparity has remained stable since 2011 after widening over the past 3 decades.^1^ The death rate in Black women has remained 40% higher than that of White women despite this population having an incidence rate equal to the mean US rate. In particular, this disparity is magnified among Black women aged younger than 50 years, with a death rate double that of White women. However, there is currently insufficient data to make specific recommendations for different racial and ethnic populations.

The current one-size-fits-all policy to screen the entire female population from a certain age may be neither fair and equitable nor optimal. In a 2021 article in *Nature*, Newsome^5^ expressed that equity in cancer screening must be improved and that one size does not fit all. Newsome^5^ also wrote that fixing the substantially lower screening rate for breast, colon, prostate, and lung cancer in the Black and Hispanic populations may dramatically improve the death rate from cancer. The current situation is an example of what happens when race and ethnicity are not considered in guidelines. This may pose a significant risk for greater harm to a group already at increased risk.^5^ To optimize the benefit of screening, risk-adapted starting ages of screening based on known and readily available risk factors, such as race and ethnicity, may be recommended. We aimed to provide evidence for risk-adapted starting age of screening by race and ethnicity.

## Methods

This cross-sectional study follows the Strengthening the Reporting of Observational Studies in Epidemiology (STROBE) reporting guideline for observational studies. Research projects involving secondary analysis of deidentified public use data sets, such as aggregated US Mortality Data and the US Surveillance, Epidemiology, and End Results (SEER) Program research data, do not require prior institutional review board approval or informed consent per the National Institute of Health Office of Human Subjects Research and 45 CFR §46.104(4).

We used mortality rates of invasive BC among female patients 2011 to 2020 from US Mortality Data. Causes of death data are collected and maintained by the National Center for Health Statistics,^6^ which can be analyzed with the statistical software of SEER (SEER*Stat). More than 99% of deaths in the United States are thought to be registered in this database.^7^ This is the largest geographic coverage available for such mortality rates, covering 100% of the US population. Only female patients who died during the study period (2011-2020) were included in this study. The outcome of interest was death due to invasive (not in situ) BC in female patients with any histology and at any stage (I-IV). Data on race and ethnicity were classified by data providers (not the investigators of this study) into 6 groups: Hispanic, non-Hispanic American Indian or Alaska Native, non-Hispanic Asian or Pacific Islander, non-Hispanic Black, non-Hispanic White, and unknown Hispanic origin. Female patients with unknown Hispanic origin (903 patients among 415 277 female patients with BC deaths [0.2%]) were excluded from analyses. For identification of race and ethnicity, the National Vital Statistics System mortality data rely on a proxy report provided by a funeral director.^8^ Demographic information on the death certificate, including race and ethnicity, was recorded by a funeral director, who was responsible for assuring the completion of the death certificate and registering it with state vital statistics offices. Information about cause of death was provided by the attending physician, medical examiner, or coroner.

### Statistical Analysis

We calculated the risk-adapted starting age of BC screening based on 10-year cumulative risk of BC-specific death by age and race and ethnicity. The 10-year cumulative risk of BC death is the risk (by percentage) of dying due to BC within the subsequent 10 years at each age. Age-specific 10-year cumulative risks of BC death among female patients were calculated using the following formulas:

1. The age-specific mortality rate is equal to the total BC-specific deaths among female patients at each age group (every 5 years) divided by the total number of person-years among females at that age group.
2. The 10-year cumulative mortality rate for each age was calculated from SEER data using the standard method of calculating the cumulative rate using aggregated data with 5-year intervals for age groups. For example, the 10-year cumulative rate for age 48 years was the rate for age group 45 to 49 years × 2 (for ages 48 and 49 years) + the rate for age group 50 to 54 years × 5 (for ages 50-54 years) + the rate for age group 55 to 59 years × 3 (for ages 55-57 years).
3. The 10-year cumulative risk was calculated as 1 – exp^(−10-year cumulative rate)^.

Comparing 10-year cumulative risk in each risk group with the population cumulative risk allowed the inference of race and ethnicity–adapted screening ages. Using our newly developed method, we could provide the age at which individuals with a certain race or ethnicity reached a similar level of risk of BC death to that of an individual aged 50 years in the general population when this is the most commonly recommended age of first screening in guidelines. We provided the same outcomes for benchmark mass screening starting ages of 40 and 45 years given that they have also been mentioned in the guidelines as starting ages of BC screening. We previously developed and used this method of calculating risk-adapted starting ages of cancers for other cancer risk factors, such as parity, age at first birth, family history of cancer, and personal history of diabetes.^9,10,11,12,13^

To exclude potential confounding by death due to COVID-19 on BC mortality by race and ethnicity, we conducted a sensitivity analysis using BC mortality in the total US population in 2011 to 2019 instead of 2011 to 2020. Data analyses were conducted in February 2023 on the latest available mortality data in the SEER*Stat program, which were obtained in February 2023. SEER*Stat statistical software version 8.4.0.1 and Microsoft Excel 2016 were used for data analyses and figure creation.

## Results

There were BC-specific deaths among 415 277 female patients (1880 American Indian or Alaska Native [0.5%], 12 086 Asian or Pacific Islander [2.9%], 62 695 Black [15.1%], 28 747 Hispanic [6.9%], and 309 869 White [74.6%]; 115 214 patients died before age 60 years [27.7%]) at any age in the US between 2011 and 2020. In the study period, 1.4% of reported US deaths among females had been assigned to the rubric for ill-defined or unknown causes (1.0% of deaths among Asian, 1.1% of deaths among Hispanic, and 1.4% of deaths among Black, American Indian or Alaska Native, and White females).

BC-specific mortality before age 50 years varied substantially by race and ethnicity. For instance, the BC mortality rate per 100 000 person-years for ages 40 to 49 years among Black females (27 deaths) was substantially higher than the mean rates among total US females (15 deaths), White females (15 deaths), and females of other races or ethnicities (11 deaths among American Indian or Alaska Native, 11 deaths among Asian or Pacific Islander, and 11 deaths among Hispanic females). The starting age of screening varied considerably by race and ethnicity group, ranging from 8 years earlier for Black females to 11 years later for Asian or Pacific Islander females compared with the benchmark starting age of population screening.

### Starting Age of Mass Screening at Various Ages

For each benchmark starting age of BC screening in the population, we calculated a risk threshold. Different race and ethnicity groups reached this risk threshold at different ages. If it were to start at age 50 years for the entire female population with a mean 10-year cumulative risk of BC death of 0.329%, Black females would reach this risk threshold at age 42 years, non-Hispanic White females at age 51 years, American Indian and Alaska Native and Hispanic females at age 57 years, and Asian and Pacific Islander females at age 61 years (Figure and Table). If BC screening was supposed to start at age 45 years for all females with mean 10-year cumulative risk of 0.235%, Black females would reach this risk threshold level at age 38 years, non-Hispanic White females at age 46 years, Hispanic females at age 49 years, Asian and Pacific Islander females at age 50 years, and American Indian or Alaska Native females at age 51 years (Figure and Table). If BC screening was supposed to start at age 40 years for the entire female population with a mean 10-year cumulative risk of 0.154%, Black females would reach this risk threshold level at age 34 years, White females at age 41 years, Hispanic females at age 43 years, and American Indian or Alaska Native and Asian or Pacific Islander females at age 43 years (Table).

**Figure.**
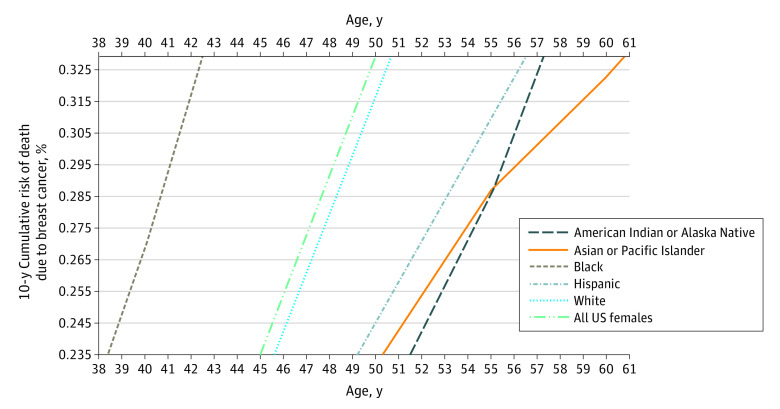
10-Year Cumulative Risk of Death Due to Breast Cancer Among Female Patients, 2011-2020 This chart is based on nationwide clinical data without any modeling (slopes are risk slopes in clinical US mortality data). Lower horizontal axis indicates population risk at age 45 years; upper horizontal axis, population risk when mass screening starts at age 50 years.

**Table.** Race and Ethnicity–Adapted Starting Age of Breast Cancer Screening

Ethnicity	Race	Starting age of screening, y		
All	Entire US population	40	45	50
Hispanic	Any	43	49	57
Not Hispanic	American Indian or Alaska Native	44	51	57
	Asian or Pacific Islander	44	50	61
	Black	34	38	42^a^
	White	41	46	51
Population risk level, %	NA	0.154	0.235	0.329

Abbreviation: NA, not applicable.

^a^ Example: If breast cancer screening was supposed to start at a benchmark age of 50 years for the entire female population with a mean 10-year cumulative risk of breast cancer death of 0.329%, Black females reached this risk threshold level of 0.329% at age 42 years. Therefore, their screening could be conducted 8 years earlier than the recommended age for the general population.

### BC Mortality Before COVID-19

Results of our sensitivity analysis using BC mortality in the total US population in 2011 to 2019 (ie, before the COVID-19 era) did not change our main finding for Black females. We still found that this population reached the respective thresholds at age 42 years when the recommendation was age 50 years, age 38 years when the recommended age was 45 years, and age 34 years when the recommendation for the general female population was age 40 years.

## Discussion

In this large, population-based cross-sectional study, we provided an evidence-based recommendation for race and ethnicity–adapted starting ages of BC screening. In general, Black females had an increased risk of dying due to early-onset BC and so could be screened up to 8 years earlier than the recommended starting age of 50 years, whereas Asian and Pacific Islander females could be screened at an older age (ie, 61 years) than the recommended staring age of screening. Exclusion of deaths in year 2020 from our study period to avoid potential confounding on BC mortality of deaths due to the COVID-19 pandemic did not change our main findings for Black females.

We also provided race and ethnicity–adapted starting ages of BC screening for other benchmark starting ages (40 and 45 years) given that mammography-screening guidelines have different recommended starting ages, ranging from age 40 to 50 years. For instance, the American Cancer Society recommends that women at mean risk levels should start annual mammography screening from age 45 years but have the opportunity to start from age 40 years.^14^ The American College of Obstetricians and Gynecologists recommends mammography in women from age 40 years and starting screening no later than age 50 years.^15^

This study did not consider the harms associated with screening, but a sophisticated simulation modeling study^16^ that considered these harms estimated that biennial mammograms for Black women starting at age 40 years would be associated with a 57% decrease in the gap in BC mortality in this populations compared with White women. Although Black women may experience disproportionate rates of false positives because of breast density, the added risk of false positives from earlier screenings may be balanced by the benefits associated with earlier BC detection in this group. To decrease the risk of false positives, one may use different screening modalities more suitable for individuals with higher breast density. Our study does not recommend any specific screening modality or interval. Our less sophisticated and more tangible study also supports earlier screening of Black females, starting from age 42 years. Additionally, we could provide race and ethnicity–adapted starting ages for other races and ethnicities in addition to Black females, which were lacking.

We believe that when fairness and optimization of resource allocation to reduce BC mortality is the aim of BC screening, health policy makers should pursue equity not just equality. Equality in the context of BC screening means that everyone is screened from the same age regardless of risk level. On the other hand, equity or risk-adapted screening means that everyone is provided screening according to their individual risk level. We believe that a fair and risk-adapted screening program may also be associated with optimized resource allocation.

The US Preventive Services Task Force recommends that women aged 40 to 49 years make an individual decision regarding screening after discussing risks and benefits of screening with their primary care practitioners. However, practicitioners would need evidence-based information on race and ethnicity–stratified starting ages of screening when individuals from minority race and ethnicity populations come to them for screening consultation.

This study used the most recent and largest nationwide cancer mortality data available in the US, with very high completeness and low rates of ill-defined causes of death, which may indirectly indicate high quality in cause-of-death data. A major strength of this study was the focus on BC death vs solely incidence. Many incidence-based calculators that seek to inform screening recommendations fail to explicitly capture increased mortality rates experienced by Black women despite slightly lower to equivalent incidence compared with White women. In a worst-case scenario, if most of the increased mortality at younger ages in Black women was due to fast-growing triple-negative cancers that were detected between screens among regular screeners, earlier screening without decreasing the interval between screenings would not be expected to decrease mortality in that subpopulation. However, earlier screening could be effective if slower-growing, screenable BCs were also occurring in Black women at younger ages. Therefore, clinical trials may be warranted to investigate whether changing screening guidelines may alter the trajectory of the disease and have a population impact. Although a previous study^17^ found that the percentage of triple-negative BC among Black females was approximately 15% compared with approximately 7% in White females and the worst-case scenario in which most BC cancers in Black females are of the fast-growing triple-negative type is not found clinically, this study provides information that may be needed to design such a trial. Another strength of this study was the provision of risk-adapted starting ages of BC screening for major race and ethnicity groups in the US, including American Indian and Alaska Native, Asian and Pacific Islander, Black, Hispanic, and White females.

### Limitations

This study has several limitations. The higher mortality in Black females may be associated with their lower level of access to screening and treatment. While this could be true for BC mortality in general, this cannot justify the higher mortality of early-onset BC in Black females younger than the recommended age of mass screening. This suggests that an earlier screening of Black females may still be associated with reduced early-onset disparity. BC mortality depends on many factors, such as differences in distribution of breast size and density, quality of screening tests, host tumor microenvironment, treatment access and quality, competing mortality, distribution of phenotype prevalence of BC, tumor grading and staging at diagnosis, initiation of BC treatment, type of treatment received, barriers to health care access, poverty level, biological and genetic differences in tumors, and prevalence of risk factors associated with the periods during and after cancer treatment, and other factors. However, making everything too complex may lead to doing nothing. One can try to start from a first scientific step (like race and ethnicity–adapted screening) and evaluate and build more complex steps along the way and reevaluate. Otherwise, the racial and ethnic disparity of early-onset BC mortality may continue or even widen again.

One should also note that regardless of race and ethnicity, cancers occurring in young female patients are inherently more aggressive. Triple-negative cancers play a role; however, other subtypes may also be aggressive. For fast-growing cancers, only lowering the mammography screening age may be associated with a less effective intervention due to aggressive interval cancers. Therefore, screening in younger females may need to be more sensitive and more frequent. However, a study^16^ found that the increased risk of false positives from earlier screenings may be balanced by the benefits of earlier BC detection, at least in young Black females between ages 40 and 50 years. This is the only racial or ethnic group in our study that needed an earlier screening.

Our analysis could not consider the competing risk of death from non-BC deaths owing to limitations in aggregated data, although this should not have substantial influence around age 50 years or younger, which was the focus of our study. No stratified analyses by molecular subtype, stage, or treatment were conducted owing to limited data on treatment or limited necessity of such analyses. The outcome in this study was BC-specific mortality, which considers only life-threatening types of this disease. The intention of this study was to provide a race and ethnicity–specific age recommendation for initiating BC screening. Given that current widely used screening modalities are able and intended to detect all invasive BC types and are not specific for any particular histology or stage, no analysis was needed by molecular subtype or stage.

The risk level found for cancer death is known to be associated with mode of detection. US Mortality Data includes a mix of female patients with no screening, infrequent screening, or regular screening prior to diagnosis, and this may be associated with racial and ethnic differences in risk of mortality. This is an inherent issue that could not be fixed in this database given that there were no data for mode of detection or past screening history. However, given that there is currently no consensus about BC screening before age 50 years, the conclusion from mortality before age 50 years in Black females, which was the focus of our study, may not have been substantially affected by this issue.

In brief, although there are more risk factors that could be used for a more optimal risk-adapted screening, the limited availability of comprehensive large, population-based, individual long-term data has always been a barrier. However, changing guidelines based on readily available risk factors, such as race and ethnicity, is possible and may be the first, yet important step toward a personalized and fair screening program.

## Conclusions

Using the latest available BC mortality information from a large, nationwide database, this cross-sectional study provided evidence-based race and ethnicity–specific starting ages of BC screening. These had substantial variations, ranging from 8 years earlier in Black females to 11 years later in Asian and Pacific Islander females compared with what is recommended. To prevent mortality due to early-onset BC before the recommended age of mass screening, the starting age for the general population could be decreased from 50 to 45 or 40 years like what is done for colorectal cancer screening in the US. However, this would come with a heavy burden to medical resources and a possible increase in potential harms associated with early screening in women at low risk before age 50 years. Instead, health policy makers may consider the alternative, risk-adapted approach in which individuals, such as Black females, who are at high risk are screened earlier. If the cost of more intensive screening in individuals at high risk matters and if the US seeks to generate guidelines that are associated with equity in mortality risk across racial and ethnic groups, the initial screening may be postponed in individuals, such as non–Hispanic Asian and Pacific Islander females, at low risk. This may decrease potential harms associated with unnecessary screenings in this population. This may be an important step toward a more optimized, equitable, and personalized BC screening and may help mitigate the current long-standing disparity of early-onset BC mortality in populations, especially Black females, at increased risk.

## Notes

Supplement. Data Sharing Statement
