# Supplementary material for: Race and Ethnicity–Adjusted Age Recommendation for Initiating Breast Cancer Screening
Source: JAMA Netw Open. 2023 Apr 19;6(4):e238893. doi: 10.1001/jamanetworkopen.2023.8893 (PMC10116360; doi:10.1001/jamanetworkopen.2023.8893)
Supplement: Supplement. — Data Sharing Statement [file jamanetwopen-e238893-s001.pdf]

Data Sharing Statement

Chen. Race and Ethnicity–Adjusted Age Recommendation for Initiating Breast Cancer Screening. *JAMA Netw Open*. Published online April 19, 2023. doi:10.1001/jamanetworkopen.2023.8893

Data
Data available: No

Additional Information
Explanation for why data not available: SEER data is publicly available to researchers at https://seer.cancer.gov/data/access.html. No further data sharing is needed/allowed.

© 2023 Chen T et al. *JAMA Network Open*.
